# A local optimization framework for addressing conservation conflicts in mosaic ecosystems

**DOI:** 10.1371/journal.pone.0217812

**Published:** 2019-05-31

**Authors:** Shane Nowack, Chris T. Bauch, Madhur Anand

**Affiliations:** 1 School of Environmental Sciences, University of Guelph, Guelph, Ontario, Canada; 2 Department of Applied Mathematics, University of Waterloo, Waterloo, Ontario, Canada; Institute of Botany of the Czech Academy of Sciences, CZECH REPUBLIC

## Abstract

An effective strategy to resolve conservation conflicts on lands outside of nature reserves is to consider the spatial arrangement of agricultural and native vegetation parcels such that the ecological value of the landscape is improved without reducing the amount of land used for agricultural production. Global optimization methods have been used to identify the best spatial arrangement of land parcels for a given project goal, but these methods are not designed to provide pathways to reach the optimum from the initial landscape. Here we describe how local search algorithms can be used to develop land parcel rearrangement pathways to obtain a landscape that sustains greater species richness than the initial landscape without changing the amount of land used for agricultural production. To demonstrate how the local optimization framework can be applied, an ecological setting based on a forest-grassland mosaic ecosystem in Rio Grande do Sul, Brazil was constructed. Plant samples collected from this region were used to construct species area curves. Multiple locally optimal solutions that improved the modeled species richness of the landscape almost to globally optimal levels were identified. To support the results, the algorithm was also applied to a 306,250 ha forest-grassland region of Rio Grande do Sul. The case study results suggested that conservation polices solely based on landowners satisfying a legal reserve percentage on their property should be revised to consider landscape-level connectivity. Providing multiple possible solutions for landscape configurations using local optimization methods may improve managerial flexibility for decision-makers, compared to global optimization approaches providing a single solution. Furthermore, the algorithm details the parcel exchange pathways that are required to reach the optimal land state. We conclude that local and global optimization approaches can be used in combination to improve land use decision-making for conservation, in mosaic ecosystems as well as other terrestrial ecosystems.

## Introduction

The introduction of sustainable land-use practices to regional landscapes often results in conflicts between the agricultural sector and conservation [1–3]. Allocating more land for conservation may have adverse effects on the livelihood of human inhabitants (reduced economic gains and less land for food production [4–5]), whereas allocating more land for agricultural purposes diminishes natural ecosystems [3,6,7], and reduces species biodiversity [8–10], which has far-reaching and irreversible effects. In the words of Sunderlin *et al*. [7] "biodiversity functions as a ‘genetic library’ [11] that supports the improvement of existing crops, introduction of new crops, and the creation of medicines and pharmaceuticals”.

Scientific communities have developed several conservation approaches that are focused on the establishment and management of nature reserves [12–13]. In fact, an entire subfield of ecology, systematic conservation planning [14], has emerged as a research area that is devoted to identifying the optimal location and design of reserves, and several pieces of systematic planning software, such as Marxan and Zonation [15], have been developed to aid in the reserve-site selection process. However, due to a growing global population that continues to drive the demand for land for agricultural purposes higher, conservation strategies outside of the creation of nature reserves must also be developed [15]. Polasky et al. [16] and others [17–20] have investigated optimal spatial arrangements for multi-use landscapes found outside nature reserves (working lands), but have focused on the nontrivial problem of obtaining globally optimal solutions, i.e., a spatial arrangement that satisfies the project objective better than any other arrangement. While identifying globally optimal solutions can inform decision-makers of what the best-case landscape should be, these methods are not designed to provide guidance regarding the implementation process [21], or alternatives to the best-case scenario. Budgetary constraints and re-establishment times of native vegetation that inherently accompany land use changes will result in a rearrangement process that takes decades to transition from the initial landscape to a landscape with improved conservation value. Thus, identifying a flexible landscape rearrangement schedule equipped to handle these challenges in a way that ensures the persistence of ecological and evolutionary processes throughout the transition period, is of utmost importance [21].

Here we introduce a local optimization method that is intended to complement global approaches by focusing on how to approach the desired landscape in a strategic manner that satisfies project objectives. The objective of local optimization is to identify solutions that are better than any other nearby solutions, whereas global optimization is intended to identify the solution that is better than all solutions. The new methodology is described in the context of landscapes possessing the attributes of a biodiversity hotspot in need of a land management strategy: the *Araucaria-Campos* forest-grassland mosaics found in the southern part of the Atlantic forest biome in Rio Grande do Sul, Brazil [22–24]. It has been argued that the Atlantic forest biome supports "one of the highest degrees of species richness and rates of endemism on the planet [25]”. A forest-grassland mosaic is a landscape consisting of forested lands and grasslands that occur roughly in equal proportions, and where it is unclear whether the natural state was either forest or grassland [26]. The *Campos* grasslands are at least twice as diverse as the forested lands in these mosaics [27–28], and more diverse than any grasslands in the world [24]. However, the *Campos* grasslands are shrinking due to changing land-use patterns, notably an expansion of agriculture and silviculture [24,29–32]. In contrast, the remaining *Araucaria* forest is currently well protected, although this has not always been the case; this can be evidenced by the fact that more than 80% of the Atlantic forest patches are less than 50 hectares in area [33].

In 2012 the Brazilian Forest Code was updated in an effort to aid in ecosystem conservation. In addition to the requirement that individual landowners maintain a legal reserve—a fixed percentage of land set aside for the growth of native vegetation—several new articles were also included [34]. For example, the environmental reserve quota (Portuguese acronym, CRA) stipulates that any landowner not in compliance with the Forest Code’s legal reserve can pay a different landowner with a surplus of natural vegetation to not convert their native vegetation to agricultural land [34]. The only stipulation is that the trade must be between properties that are in the same biome, and preferably in the same state. The long-term consequences of these new policies are not yet clear.

Motivated by the example of the CRA, our objective is to develop a framework based on local optimization to determine land state configurations that improve species richness in mosaic ecosystems under parcel exchange mechanisms. The algorithm starts from a landscape consisting of a forest-grassland mosaic and agricultural lands and demonstrates a local optimization approach to (i) identify multiple, alternative landscape patterns that improve species biodiversity without reducing the total amount of land for agricultural activities and (ii) provide a step-by-step pathway for the parcel exchanges leading to the desired land use configuration. The attributes of a forest-grassland mosaic were modeled by constructing species-area curves from grassland samples collected in Aparados da Serra National Park, Rio Grande do Sul, Brazil (G. Overbeck, unpublished data), and applying previously published relationships between the forest biodiversity and grassland biodiversity [27–28] in this region.

## Materials and methods

### The model

Each hypothetical landscape that was optimized was assumed to consist of an equal number of *parcels* that were entirely agriculture (Ag), forest (F), or grassland (G) (the three land states that dominate the *Araucaria-Campos* forest-grassland mosaics). The equal-parcel assumption derives from: (i) the constraint that the total amount of agricultural lands cannot be reduced (to address the conservation conflict issue discussed in the introduction), and (ii) the definition of a forest-grassland mosaic (i.e., forest and grassland occur in roughly equal proportions [26]). However, our optimization framework also works for a non-equal distribution of parcel types.

If parcel *i* is in land state *j*, then we write

$$x_{i,j}=1,$$ (1)

and if parcel *i* is not in land state *j*, then we write

$$x_{i,j}=0,$$ (2)

where *i* ∈ {1, …, *M*}, *M* is the total number of parcels on the landscape, and *j* ∈ {*Ag*, *F*, *G*}. It follows that

$$x_{i,Ag}+x_{i,F}+x_{i,G}=1$$ (3)

must be satisfied for each *i* ∈ {1, …, *M*}.

On every landscape we collect parcels into *Q* patches, where 1 ≤ *Q* ≤ *M*. A *patch* is defined as a collection of *k* parcels that are in the same land state, and that are von Neumann neighbors. The von Neumann neighbors of a given parcel consist of all parcels that are directly above, below, to the right, or to the left of it [35].

**Fig 1:**
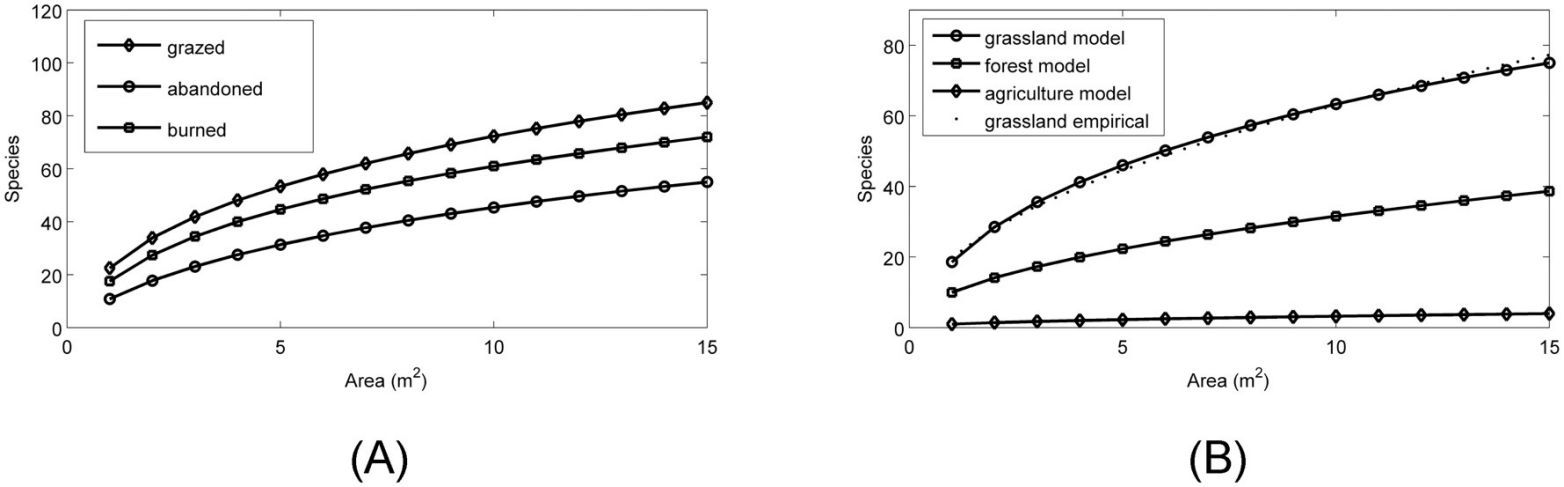
Species-area relationships (SARs). (A) Empirical SARs for the different subcategories of grassland that were sampled in Aparados da Serra National Park, Rio Grande do Sul, Brazil. (B) Grassland SAR (circle), forest SAR (square), and agriculture SAR (diamond) fitted to empirical data and used to parameterize the model. The arithmetic mean of the empirical SARs (dotted) of the subcategories of grassland (shown in (A)) is shown for comparison purposes. See *Species-area relationships* subsection for details on data sources.

Next, let *B*_*L*_ represent the number of species in a patch *L*.

$$B_{L}=\sum_{k|P_{k}\in D_{L}}c_{j}{\left(\sum_{k|P_{k}\in D_{L}}Ax_{k,j}\right)}^{z_{j}}$$ (4)

$$=\sum_{k|P_{k}\in D_{L}}c_{j}{\left(\sum_{k|P_{k}\in D_{L}}A\right)}^{z_{j}}$$ (5)

$$=\sum_{k|P_{k}\in D_{L}}c_{j}A_{L}^{z_{j}},$$ (6)

where *A* represents the area of a single square parcel, *A*_*L*_ is the area of patch *L*, *P*_*k*_ represents parcel *k*, and *D*_*L*_ is the set of all parcels that are in patch *L*. Here $c_{j}A_{L}^{z_{j}}$ represents the power-law species-area relationship (SAR) for a patch in vegetative state *j*. The parameters that govern the power-law, *c*_*j*_ and *z*_*j*_, were fit using empirical data collected from Aparados da Serra, Rio Grande do Sul, Brazil (see Table 1, Fig 1, S1 and S2 Files, and the following section for further details).

**Table 1:** Parameter definitions.

Quantity	Description
${\text{SAR}}_{\text{G}}={\text{c}}_{\text{G}}{\left(\text{Area}\;\text{of}\;\text{patch}\right)}^{z_{G}}$	Species-area relationship for grasslands (species/m^2^)
${\text{SAR}}_{\text{F}}={\text{c}}_{\text{F}}{\left(\text{Area}\;\text{of}\;\text{patch}\right)}^{z_{F}}$	Species-area relationship for forested lands (species/m^2^)
${\text{SAR}}_{\text{Ag}}={\text{c}}_{\text{Ag}}{\left(\text{Area}\;\text{of}\;\text{patch}\right)}^{z_{Ag}}$	Species-area relationship for agricultural lands (species/m^2^)
*c*_*G*_ = 19.95	Fitted to empirical data
*c*_*F*_ = 0.5*c*_*G*_	Extrapolated from grassland data and Overbeck *et al*. (2006)
*c*_*Ag*_ = 0.05*c*_*G*_	Model assumption
*z*_*G*_ = 0.51	Fitted to empirical data
*z*_*G*_ = *z*_*F*_ = *z*_*Ag*_	Model assumption

Our objective was to optimize species richness under a parcel exchanging process similar to what occurs under the CRA, using SARs to estimate species number. The term “parcel exchange” is used to refer to an exchange of land states between two parcels. The possible exchanges are one grassland parcel can be exchanged with one agriculture parcel (this means one grassland parcel is changed to an agriculture parcel, and one agriculture parcel is changed to a grassland parcel), one forest parcel exchanged with one agriculture parcel, and one grassland parcel exchanged with one forest parcel. To generate a relatively simple objective function to optimize we made the following assumptions: (i) the relationship between number of species and patch size is captured by SARs (patches of greater area have more species); (ii) the total land area of each land state type remains unchanged (due to exchanging). One of the simplest functional forms that satisfies these assumptions and captures species richness in a mosaic is:

$$B=\sum_{L=1}^{Q}B_{L}$$ (7)

We refer to *B* as the *mosaic richness index*. To ensure that a set of N connected parcels has a higher mosaic richness index than N disconnected parcels, the patch SARs in Eqs (4)–(6) have been weighted by the number of parcels in the patch. For instance, if we did not sum over the number of parcels in Eq (6), then the algorithm would incorrectly identify two disconnected patches of equal area A as having more species than one larger patch of area 2A.

More sophisticated functional forms could incorporate elements such as patch architecture (i.e., the distribution of patches across space), species dispersal mechanisms, and dispersal kernels, and would be compatible with our local optimization framework. For example, an objective function that accounts for patch architecture, *B*′, could be formed by adding a patch architecture term to Eq (7),

$$B^{'}=w\sum_{L=1}^{Q}B_{L}+{(1-w)P}_{A}$$ (8)

where *P*_*A*_ represents the effect of patch architecture and *w* is a weighting factor that determines the relative importance of within-patch connectivity (*B*_*L*_) versus patch architecture. The patch architecture term will be determined by the specific ecosystem properties. Several ways to model patch architecture have been discussed in the literature (see [36]). Possible forms of *P*_*A*_ are listed in Eqs (9) and (10) below:

$$P_{A,1}=\frac{1}{\sum_{i=1}^{S}d(F_{i})}+\frac{1}{\sum_{i=1}^{T}d(G_{i})}$$ (9)

$$P_{A,2}=\frac{1}{\sum_{i=1}^{S}E(F_{i})}+\frac{1}{\sum_{i=1}^{T}E(G_{i})}$$ (10)

In Eq (9), *P*_*A*,1_ is a patch architecture term that increases the mosaic richness index of a landscape if the forest patches and/or grassland patches are closer to one another. In Eq (9) *d*(*F*_*i*_) represents the minimum distance the centroid of forest patch i is from the centroid of any other forest patch, and *d*(*G*_*i*_) represents the minimum distance the centroid of grassland patch i is from the centroid of any other grassland patch, S is the total number of forest patches, and T is the total number of grassland patches. In contrast, in Eq (10), *P*_*A*,2_ is a patch architecture term that increases the mosaic richness index of a landscape if forest and grassland patches have parcels that are adjacent to one another (since adjacent forest-grassland patches may be more desirable than adjacent forest-agriculture or grassland-agriculture patches from a forest-grassland mosaic conservation perspective). In Eq (10), *E*(*F*_*i*_) is the number of forest parcels in patch i that are adjacent to agriculture parcels and *E*(*G*_*i*_) is the number of grassland parcels in patch i that are adjacent to agriculture parcels The modeler may wish to include both of the effects in Eqs (9) and (10) into the objective function, and this can be done by replacing *P*_*A*_ with *P*_*A*,1_ + *P*_*A*,2_ in Eq (8).

Another possibility would be to include costs in the objective function. We note that if costs were included then the objective function would no longer be a mosaic richness index, and the objective would not be to maximize species diversity. For this reason we have not included costs in our model. Similar to patch architecture, there are a number of ways to incorporate costs into an objective function, and will vary from project to project. We present one intuitive way to include costs in Eq (11) below:

$$B^{''}=w\sum_{L=1}^{Q}B_{L}+(1-w)C,$$ (11)

where

$$C=\frac{1}{{G_{F}+G_{A}+F}_{G}+F_{A}+A_{F}+A_{G}},$$ (12)

and where (*G*_*F*_) is the total cost associated with changing the necessary forest parcels to grassland (along a given parcel exchange pathway), (*G*_*A*_) is the total cost associated with changing the necessary agriculture parcels to grassland, (*F*_*G*_) is the total cost associated with changing the necessary grassland parcels to forest, (*F*_*A*_) is the total cost associated with changing the necessary agriculture parcels to forest, (*A*_*F*_) is the total cost associated with changing the necessary forest parcels to agriculture, (*A*_*G*_) is the total cost associated with changing the necessary grassland parcels to agriculture, and *w* weights the relative importance of maximizing biodiversity versus minimizing costs.

In general, there is no limit to what effects can be incorporated into the objective function. The focus of this paper is on advancing land use planning activities by incorporating local optimization methods into the field, thus, we analyze the more specific form of the mosaic richness index that is defined in Eq (7) (without including costs and the patch architecture terms).

We also note that parcel connectivity can be modeled in many different ways [37], and how it is defined can vary from species to species and/or landscape to landscape. We have chosen to model connectivity as structural connectivity (contiguous parcels forming a patch) in order to rank landscapes that possess corridors of vegetation types higher than fragmented landscapes. Furthermore, the only surrogate inputs included in the objective function are species-area relationships for the native plant vegetation. Although it has been reported that the plant biodiversity has a trickle-down effect on all other biodiversity [38–39], from terrestrial animal species, to birds, to microbial species [40], using such surrogates to represent all biodiversity has been criticized [41–42]. We only consider species richness in Eqs (4)–(7), although biodiversity measures such as Shannon entropy could also be used.

### Species-area relationships

The grassland SAR data (S1 and S2 Files) originated from samples collected in 15–1 m^2^ parcels from grasslands located in Aparados da Serra National Park, Rio Grande do Sul, Brazil, (central coordinates are 29°09′20.7″ S, 50°07′13.78″W) using the Londo [43] cover scale (samples collected by G. Overbeck and colleagues). Permission to sample in the National Park was granted by the ICMBio (Instituto Chico Mendes de Conservação da Biodiversidade), which administrates the National Park sampling permits. From raw species counts, a method of Scheiner [44] was used to construct the grassland SARs (Fig 1A). The basis of the method used is to average all one-parcel, two-parcel, three-parcel, etc. combinations in order to obtain the number of distinct species that an area of a given size can sustain. The grasslands that were sampled were further subcategorized into one of three types: abandoned (cattle has access in theory, but based on composition and structure we can say that they almost do not enter), grazed (moderately grazed), or recently burned (burned approximately six months before sampling, before that abandoned). For purposes of parameterizing the model the arithmetic mean of these three SARs was used (Fig 1B; dotted). The forest SAR used to parameterize the objective function was extrapolated from previously reported findings in this region [27–28]; the species richness of the forested regions has been estimated to be half that of the grasslands, and this was represented in the model by setting *c*_*F*_ = *c*_*G*_*0*.*5*. Furthermore, since the SARs are based on plant species and most croplands are monocultures [45], *c*_*Ag*_ = *c*_*G*_*0*.*05* was assumed in the agriculture SAR. This assumption was based on the fact that *c*_*G*_ is approximately 19.95 species/m^2^ in this study, and we expect to there to be one crop species/m^2^ in an agriculture patch.

### Local optimization methodology

**Fig 2:**
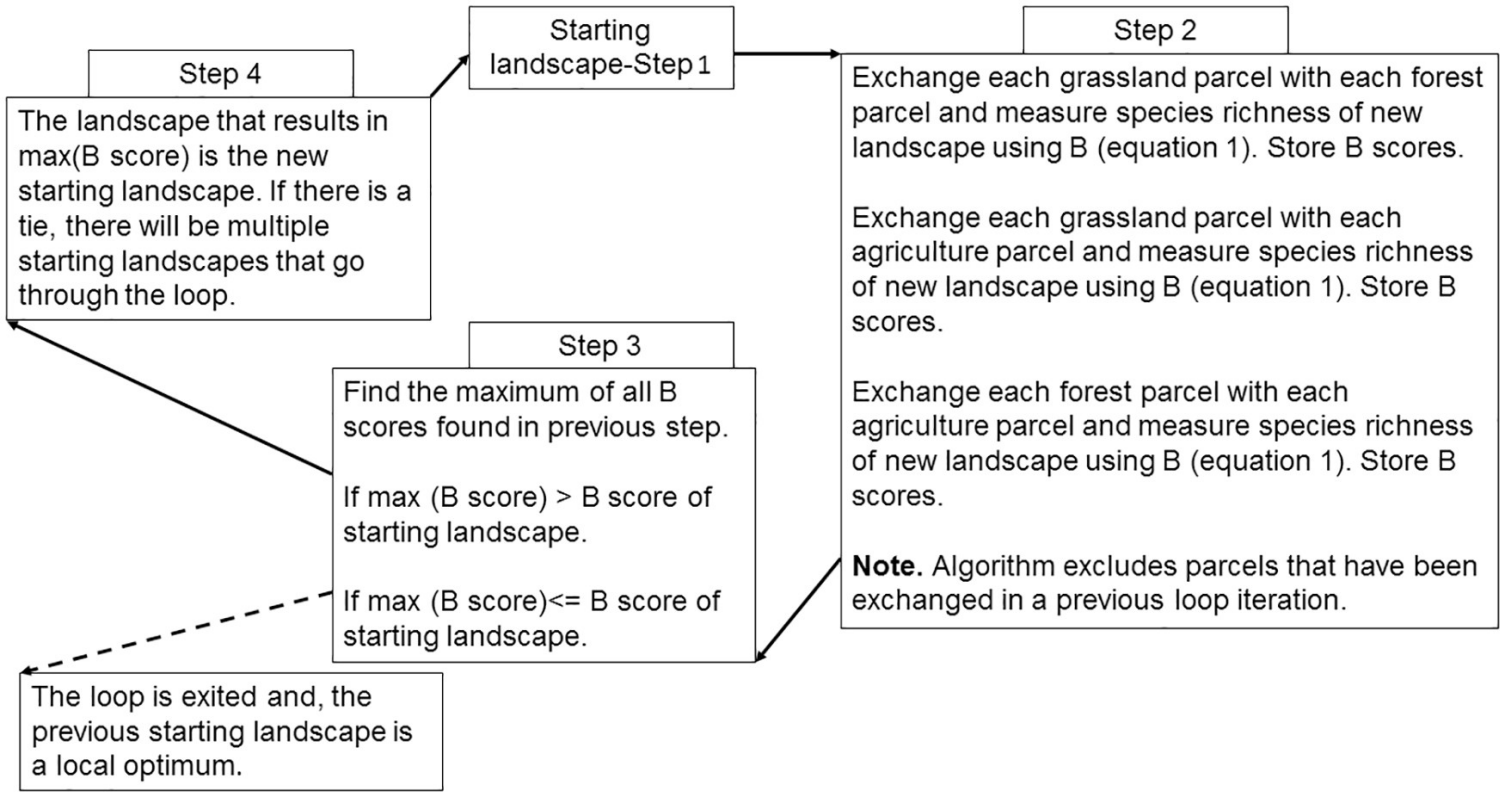
Schematic of local optimization process.

A local search algorithm (an optimization heuristic that is a sub-class of standard hill-climbing algorithms [46]) was implemented (using MATLAB; see S2 Appendix for code) to generate landscapes that locally maximize the mosaic richness index *B* from (7) subject to the constraints that the number of parcels in each land state cannot change, and the only possible parcel exchanges (PEs) are one grassland for one agriculture, one forest for one agriculture, and one grassland for one forest. In the simulations involving the hypothetical landscapes consisting of 36 parcels, the PEs can occur within a single landowner’s property, or between two different landowner’s properties. The optimization routine starts by making the one PE on the landscape that increases B the most. Given this change, the routine then picks a new PE that increases B the most. This process continues until no PE can further improve *B*. We define the end landscape as a *locally optimal landscape*. The sequence of intermediate landscapes resulting from PEs is defined as a *path*. Paths leading from the initial landscape to a locally optimal landscape are defined as *locally optimal paths*. Fig 2 summarizes the optimization algorithm.

Multiple iteration steps are likely to result in *ties*—instances in which two or more exchanges result in the highest increase in B (the mosaic richness index). When ties occur, all paths originating from the tied landscapes were followed, and resulted in the identification of multiple, locally optimal landscapes. To rank tied outcomes, we introduce two additional terms. A *minimal path* is a locally optimal path that requires the fewest number of PEs to reach a locally optimal landscape. A *dominant minimal path* is a minimal path that has a mosaic richness index at each intermediate landscape that is greater than or equal to the mosaic richness indices of intermediate landscapes that occur along any other minimal path. For initial landscapes in which at least one dominant minimal path exists, these paths will be ranked the highest, but we also will consider other paths that minimally deviate (e.g., an extra parcel exchange or a single intermediate landscape that does not have a maximal *B* value) from dominant minimal paths in order to avoid discarding reasonably good alternatives. For other initial landscapes a dominant minimal path will not exist. In these cases, locally optimal paths that minimally deviate from the notion of a dominant minimal path will be those that are included in the list of viable candidates. One final note is that the viable candidate lists considered here exclude any candidate that arose from a path in which a parcel’s vegetative state was changed more than one time. This decision was made due to practical reasons. If a sequence of land state changes is to be followed, it does not seem logical or efficient for a path that involves changing a parcel’s vegetative state more than one time to be followed.

### Description of case study region

**Fig 3:**
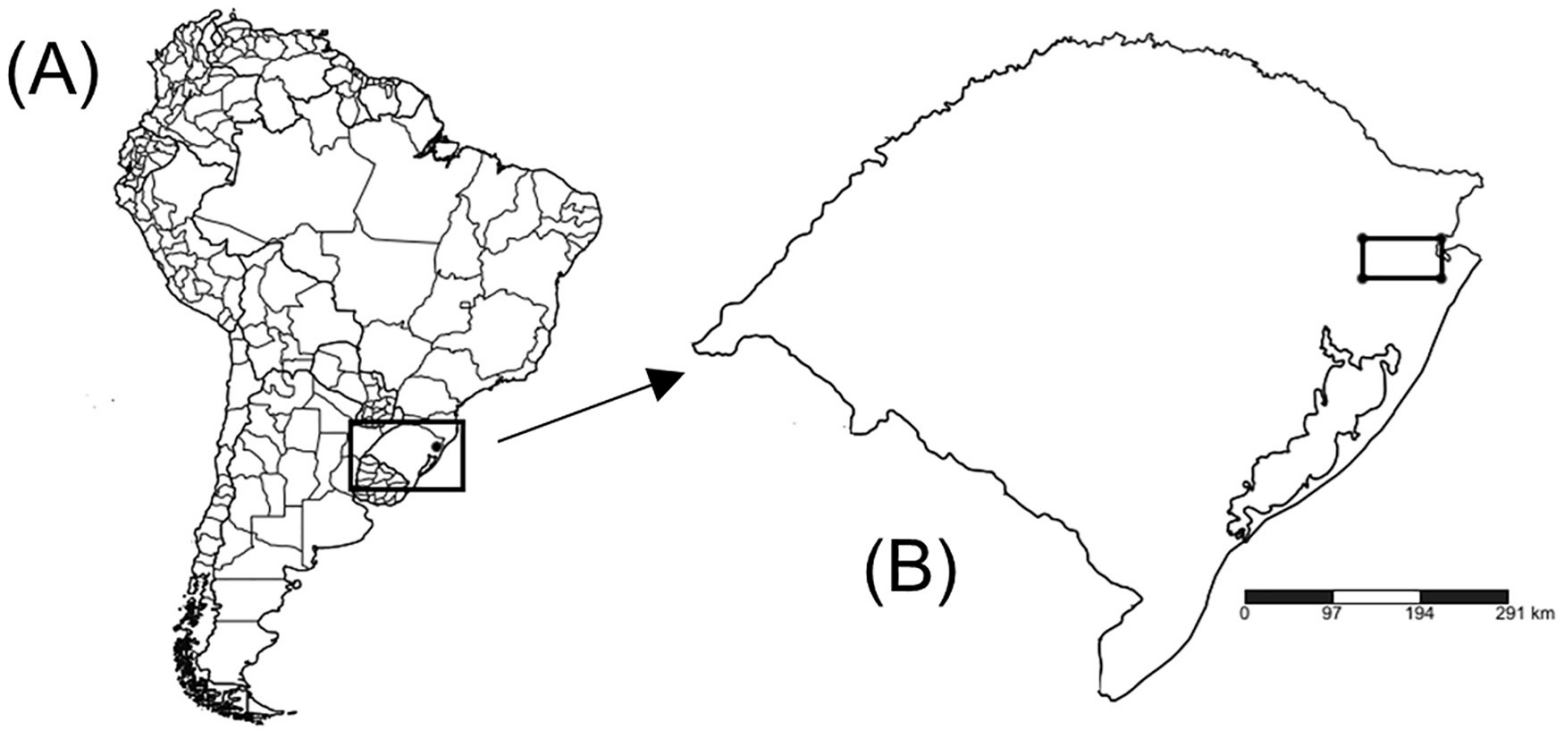
Case study region. (A) Geographical region of study area (South America with the state of Rio Grande do Sul, Brazil in rectangle). (B) Rio Grande do Sul, Brazil with case study region in rectangle.

In addition to exploring how the algorithm works on hypothetical land state configurations, we also applied the algorithm to a real-world case study region. For this case study, we used 25 landowner properties found in a rectangular 306,250 ha region (Fig 3) located near the Atlantic Forest and Pampa biome boundary of Rio Grande do Sul, Brazil between coordinates 29°07′38.6″ S, 50°54′ 22.3″W (northwest corner) and 29°31′37.2″ S, 50°07′ 11.3″W (southeast corner). The vegetative cover of the entire region was mapped in 2002 using a mosaic of Landsat 5TM and Landsat 7ETM + images [47], and a shapefile detailing this information is available at the University of Rio Grande do Sul’s Laboratory of Geoprocessing web site (www.ecologia.ufrgs.br/labgeo/). ArcGIS was used to construct a rasterized version of the shapefile and to produce a map that consists of four main land covers: urban, agriculture, forest, and grassland. Each raster is 10 ha in area and the category is assigned a land state according to the predominant land cover of that square area. See Results section for rasterized version of case study region.

We explored two different kinds of simulations in the case study region. To demonstrate the flexibility in the algorithm, one assumption from the local optimization methodology changed. Specifically, restrictions on different types of allowable parcel exchanges were explored. Recall that the simulations on the hypothetical landscapes were run without any restrictions. However, in the first case study simulation the only exchanges considered were intra-property parcel exchanges. The goal here was to investigate the landscape level effects of actions taken at the property level. In the second simulation, a CRA-type exchange was simulated by only allowing inter-property parcel exchanges. In this simulation the assumption is that one property owner would like to increase their agricultural land by an area equivalent to 10 parcels, and the other property owner is willing, for a negotiated price, to reduce their agricultural land by an area equivalent to 10 parcels. The algorithm is still making one parcel exchange per iteration, but an extra constraint is included so that the algorithm stops after 10 iterations to account for the agreed upon area.

## Results

### Multiple locally optimal landscapes

**Fig 4:**
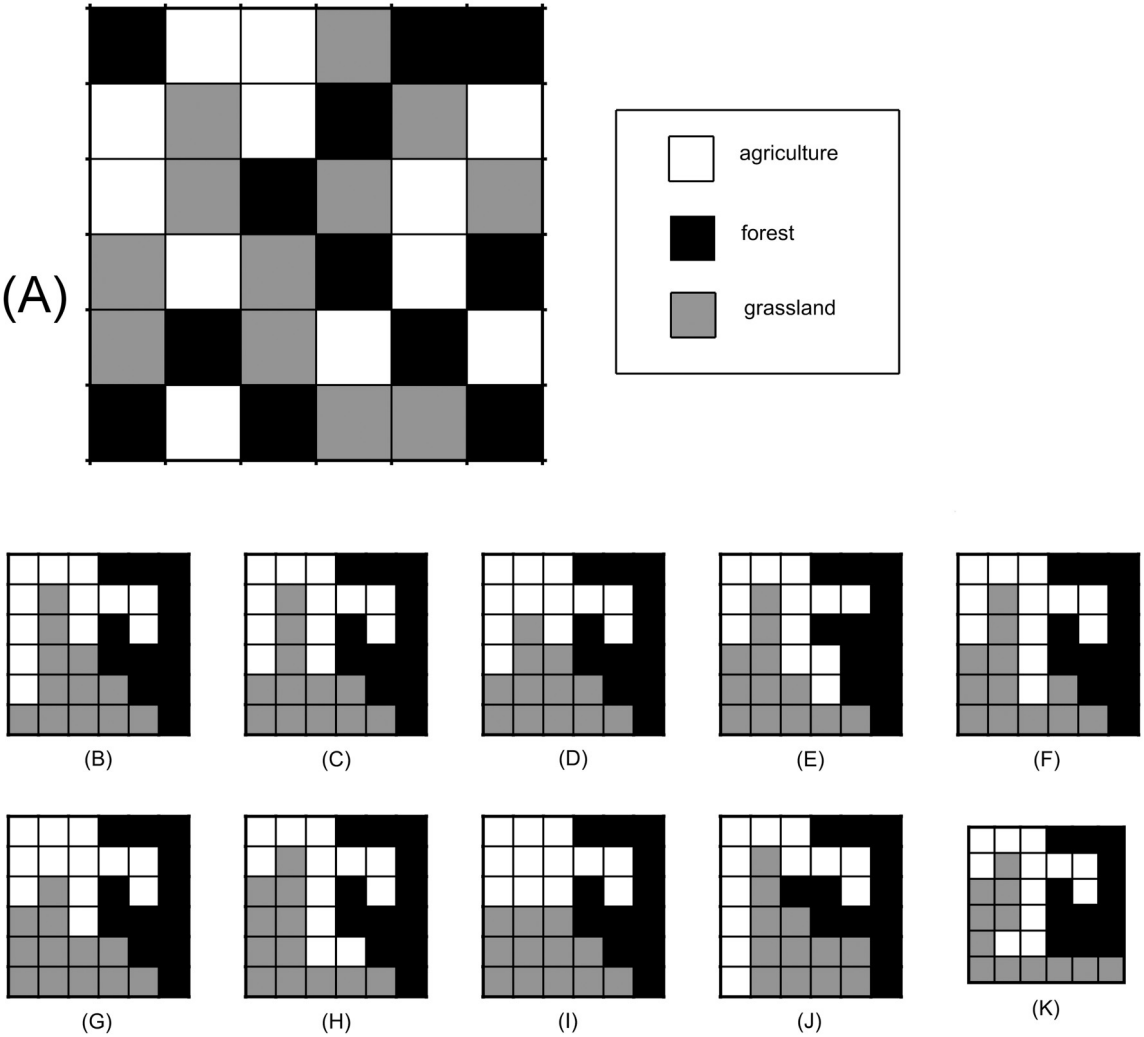
Multiple locally optimal landscapes. **Example 1: similar viable candidates**. (A) First hypothetical initial landscape. (B)-(I) All eight of the unique, locally optimal landscapes arising from (A) via dominant minimal paths (see Table 2). (J)-(K) Two of the five unique, locally optimal landscapes arising from locally optimal paths that only minimally deviated from dominant minimal paths; the corresponding paths are shown in Fig 6B and 6C, respectively, and the minimal deviations are explained in the caption of Fig 6. Collectively, subfigures (B)-(K) demonstrate that (i) the algorithm leads to multiple locally optimal landscapes, and (ii) the viable candidates shown collect the parcels of each vegetative state into a single patch, hence, these viable candidates are also global optima. Note that for this particular initial condition that all viable candidates shown are relatively similar to one another, with only subtle differences observed in their arrangements.

**Fig 5:**
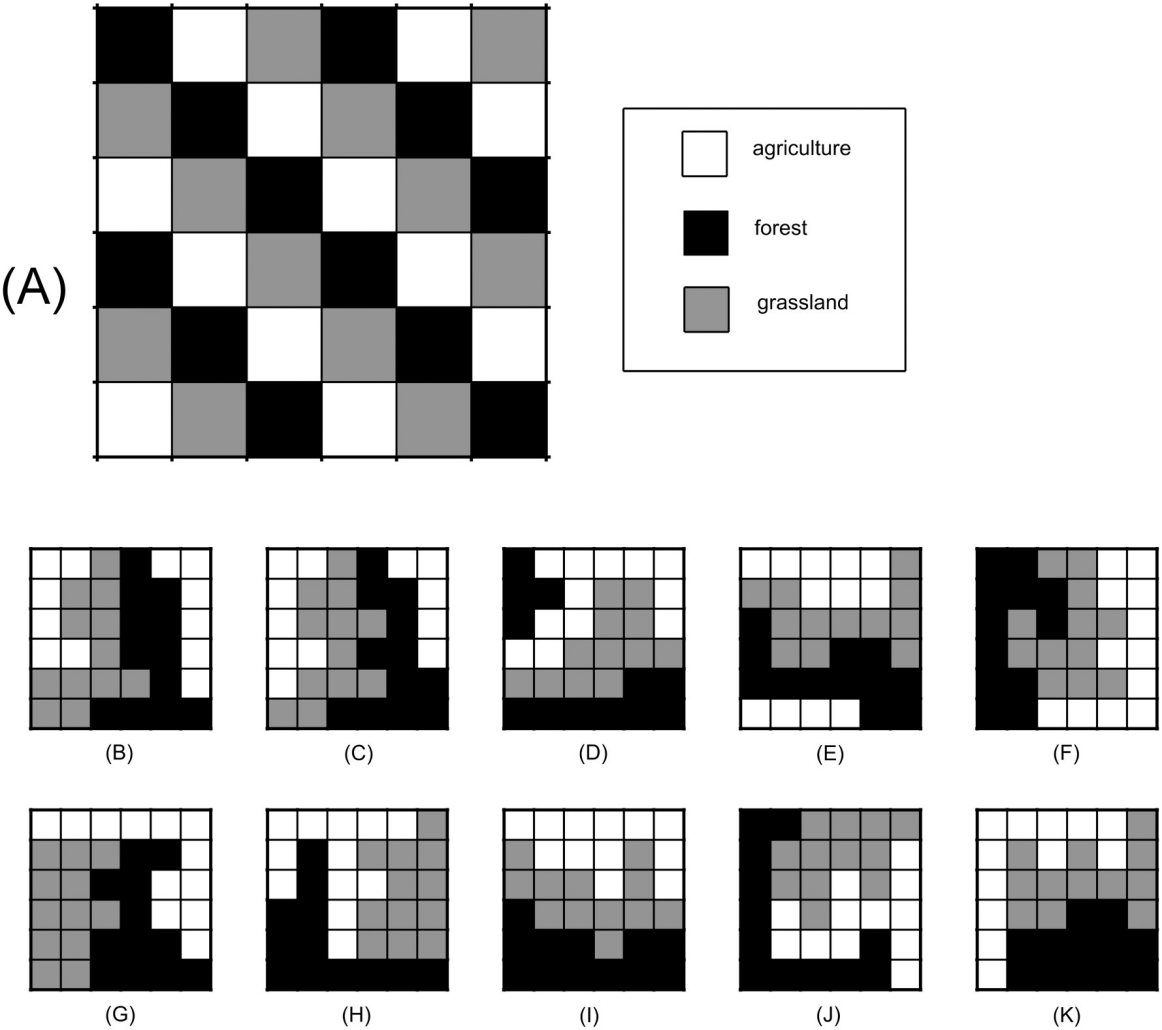
Multiple locally optimal landscapes. **Example 2: dissimilar viable candidates**. (A) Second hypothetical initial landscape. No dominant minimal paths exist for this initial landscape. These landscapes were derived from locally optimal paths that minimally deviated from the notion of a dominant minimal path (i.e., arose either from a minimal path that was not ideal, or a locally optimal path that required one more PE than an associated minimal path). (B)-(E) A selection of four of the 179 unique, locally optimal landscapes associated with the initial landscape in (A) and that are not global optima (all patches are not connected). (F)-(K) Six additional unique, locally optimal landscapes that also arose from (A). These landscapes are global optima as well (all patches are connected). Unlike (B)-(K) in Fig 4, (B)-(K) in this figure demonstrate that some initial landscapes lead to viable options that are very different from one another. The minimal deviants considered here were either derived from (i) minimal paths that were not increasing (Figs 4J and 5B), or (ii) locally optimal paths that required one additional PE, compared to that of a minimal path (Figs 4K and 5C–5K). Note that with some initial landscapes, all viable candidates are very similar (Fig 4B–4K), and with others, the viable candidates are quite different (Fig 5B–5K). Both the number of viable candidates and the differences among the candidates are dependent on how close the initial landscape is to a local optimum, and also the criteria used to define a minimal deviant.

Each hypothetical initial landscape resulted in multiple, locally optimal landscapes when the local search algorithm was applied (Figs 4 and 5). Locally optimal landscapes that were derived from either dominant minimal paths (Fig 4B–4I), or from locally optimal paths that minimally deviated from a dominant minimal path (Figs 4J, 4K and 5B–5K), comprise the list of viable candidates (see Table 2 for summary of model output related to viable candidates).

**Table 2:** Summary of simulation output related to paths arising from initial landscapes shown in Figs 4A and 5A that led to viable candidates.

Figure Number	Number of Dominant Minimal Paths (Distinct End)	Number of Minimal Deviants (Distinct End)
4	40 (8)	12 (5)
5	0 (0)	20758 (179)

From both initial landscapes (Figs 4A and 5A), the local optimization algorithm organizes the parcels of each native vegetation state into a single patch (with the exception of Fig 5D). This general patch behavior was expected due to the choice of the objective function, and the relationship between the SAR parameters and the form of the objective function (S1 Appendix). Further, we find the locally optimal landscapes shown in Figs 4B–4K and 5F–5K are also globally optimal landscapes. To understand why this is true note from Eq (6) that the mosaic richness index for a single patch with area A*N is ${Nc}_{j}{\left(A*N\right)}^{z_{j}}=c_{j}A^{z_{j}}N^{z_{j}+1}$, where *N* is the number of parcels in the patch. If that single patch is split into two patches that have *N*_1_ parcels and *N*_2_ parcels, such that *N*_1_ + *N*_2_ = *N*, the mosaic richness index is $c_{j}A^{z_{j}}{{(N}_{1}}^{z_{j}+1}+{N_{2}}^{z_{j}+1})$. From Bernoulli’s inequality [48] it can be shown that ${{{{(N}_{1}+N_{2})}^{z_{j}+1}\geq (N}_{1}}^{z_{j}+1}+{N_{2}}^{z_{j}+1})$, which shows that combining all parcels of a certain type into one patch will always yield a higher mosaic richness index than organizing them into two patches. Proof by induction is then used to show that, for a fixed number of parcels that are in a given state, fewer patches will always result in a higher mosaic richness index (see S1 Appendix for the complete proof). In general, local search algorithms will not necessarily lead to globally optimal solutions (see Fig 5B–5E; they are not global optima because either the forest or agriculture patches are not all connected), and with a more complicated objective function, identifying whether a locally optimal landscape is also globally optimal will be less trivial.

### Parcel exchange maps

**Fig 6:**
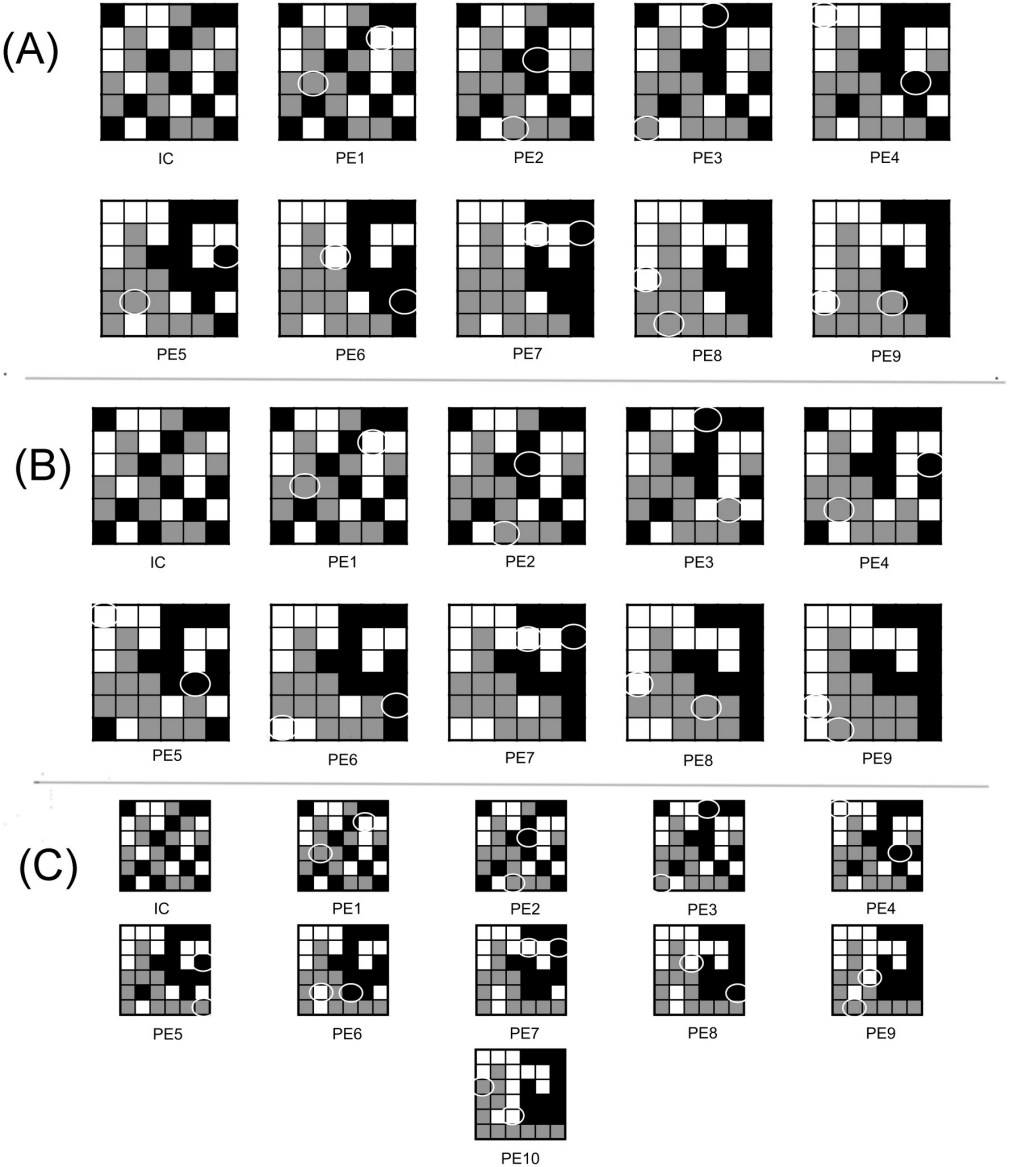
Dominant minimal paths and other locally optimal paths that minimally deviate from dominant minimal paths associated with the initial landscape shown in Fig 4A. (A) One of 40 dominant minimal paths (corresponds to locally optimal landscape shown in Fig 4B). The circles indicate parcels that were exchanged from the previous step. (B) One of 12 locally optimal paths that minimally deviated from a dominant minimal path (corresponds to locally optimal landscape shown in Fig 4J). The minimal deviation in this case is that the locally optimal path is a minimal path that is not increasing, and this can be observed in Fig 8A (dotted). (C) A second locally optimal path that is not a dominant minimal path (corresponds to locally optimal landscape shown in Fig 4K). 10 parcel exchanges were required to reach a locally optimal landscape, compared to the 9 parcel exchanges required in the paths shown in (A) and (B). The parcel exchange maps provide a rearrangement schedule, and allow decision-makers to visualize how the rearrangement process may unfold in practice.

**Fig 7:**
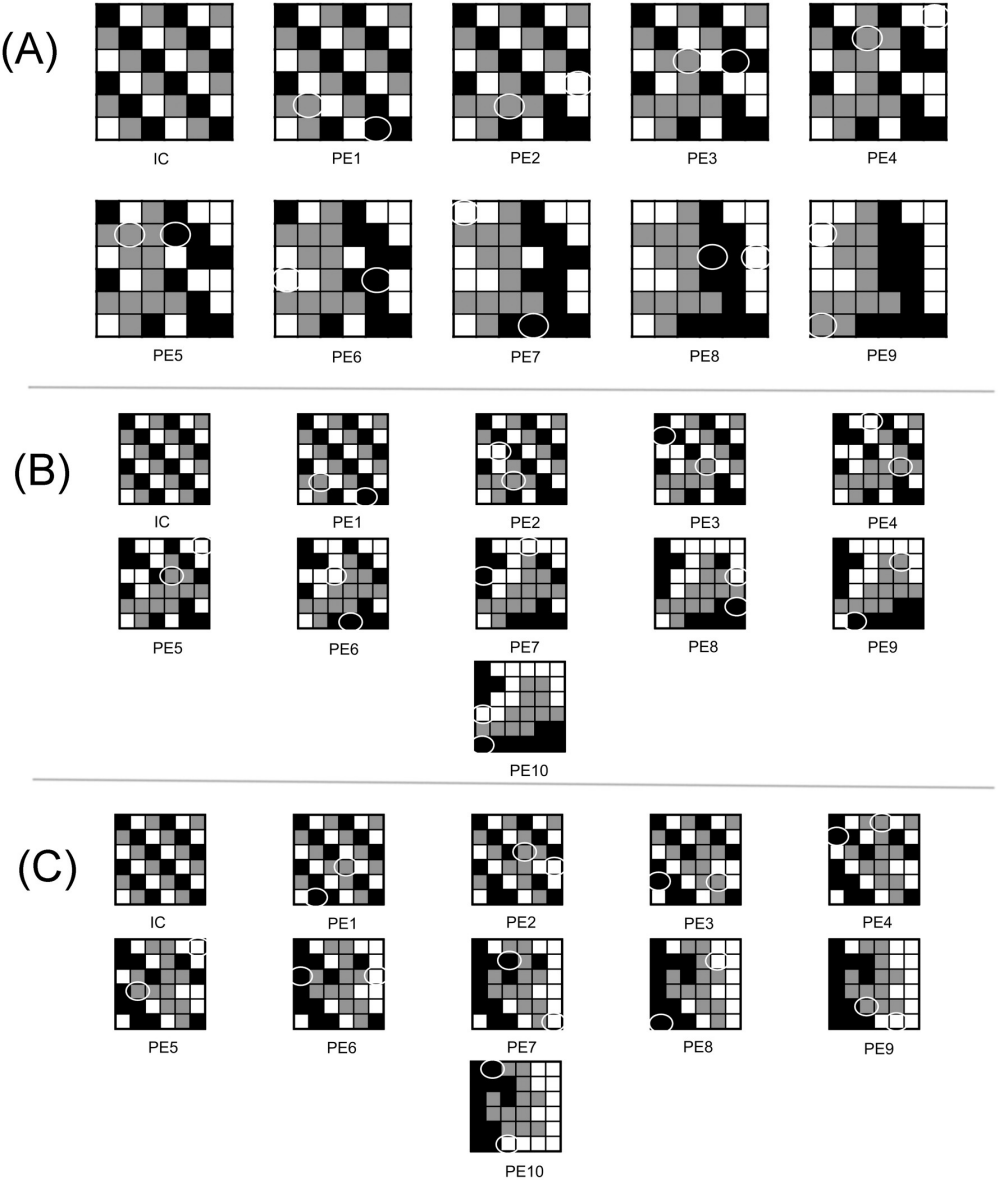
Locally optimal paths that minimally deviate from the notion of a dominant minimal path and that were derived from the initial condition shown in Fig 5A. (A) A minimal path (corresponds to the locally optimal landscape shown in Fig 5B) that does not attain the maximal mosaic richness index at the locally optimal landscape (see Fig 8B, solid), hence this minimal path is not a dominant minimal path. B) A locally optimal path (corresponds to the locally optimal landscape shown in Fig 5D) that can be excluded from the list of viable options due to poor relative species richness sustained at each intermediate landscape along the path (see Fig 8B, dotted). (C) A locally optimal path (corresponds to the locally optimal landscape shown in Fig 5F) that supports maximal diversity at each intermediate landscape along the path, relative to all other locally optimal paths that end in a globally optimal landscape (see Fig 8B, dashed), but is not a minimal path. The paths in (A) and (C) are both viable options, but is the end result of a global optima in (C) necessarily better than the local optima that requires fewer parcel exchanges (shown in (A)), which does not reach a global optima?

In addition to identifying multiple, locally optimal landscapes, the algorithm also produced parcel exchange maps (also referred to as locally optimal paths) that lead from the initial landscape to locally optimal landscapes (see Figs 6 and 7). The parcel exchange maps describe the step-wise landscape rearrangement process.

The parcel exchange maps also reveal another general property of the algorithm—one that is specific to the objective function and may be desirable from both a practical and an ecological standpoint. That is, patches of a given vegetation type grow from smaller “seed” patches through a process resembling nucleation (see Figs 6 and 7). This is desirable because natural land state patches that are derived from expansion of existing neighboring natural patches are more likely to inherit the mature, late succession species compositions of those neighboring patches, compared to vegetation generated in areas where they do not naturally occur (for instance, restored areas are reported to support less biodiversity than natural areas [49–52]).

Also, we note that the algorithm has a tendency to first connect the grassland parcels into a single patch, then the forest parcels, and then finally the agriculture parcels. The order in which the patch types are connected can be explained by the SARs (See Fig 1B), i.e., grasslands of this region sustain higher species richness than forests, which in turn sustain more species than agriculture lands. There will be instances in which this order of connectivity will be violated, and this depends on the sizes of the largest patches of a given vegetative state.

### Post-simulation analyses

**Fig 8:**
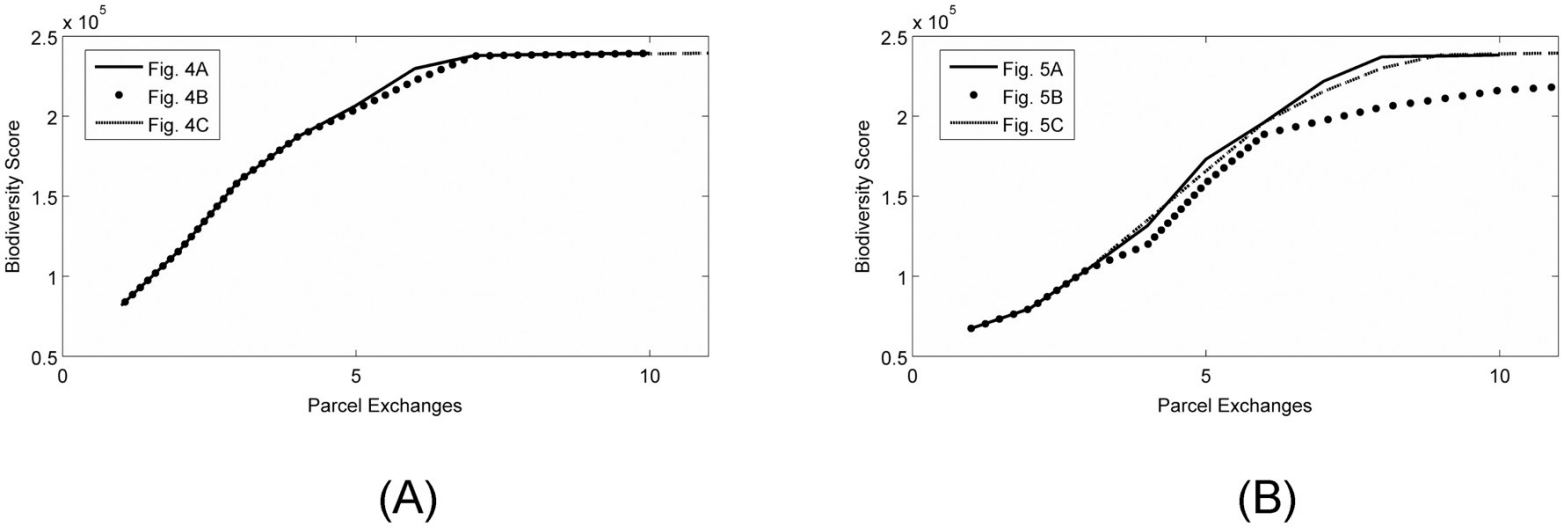
Ranking system for the viable options that arise from different initial landscapes. (A) Mosaic richness indices along the paths shown in Fig 6 that originated from the initial landscape shown in Fig 4A. The mosaic richness indices of the intermediate landscapes that occur along the three different paths are very similar, so much so that it is difficult to differentiate between the dashed line (corresponding to Fig 6C) and the solid line (corresponding to Fig 6A). Thus, for this initial condition, it is clear that several good options exist that do not correspond to dominant minimal paths (e.g., the dashed and dotted lines), and highlights the reason one may want to consider minimal deviants. (B) Mosaic richness indices along the paths shown in Fig 6 that originated from the initial landscape shown in Fig 5A. For this initial condition, the mosaic richness indices of the intermediate landscapes vary considerably along the paths. It is clear that the path shown in Fig 7B (dotted line) is not the best choice for this initial landscape. Alternatively, the paths shown in Fig 7A and 7C are comparable, but the graph supports the argument that, in this situation, maybe the path shown in Fig 7C (dashed) that leads to a global optimum is not as good as the locally optimal path shown in Fig 7A (solid).

Plots comparing the mosaic richness indices of the intermediate landscapes associated with the viable candidates (related to Figs 6 and 7) were used to hierarchically rank the locally optimal paths (Fig 8). In practice, intuitive plots such as these can be used to quantify the effects of choosing one local optimum over another, and help decision-makers explain choices to concerned parties. Furthermore, social or cultural constraints that were unable to be modeled mathematically may require decision-makers to choose a path other than highest ranked option. If the initial landscape yields several locally optimal paths that are closely ranked (Fig 8A), decision-makers have more flexibility in choosing lower-ranked options while still being able to meet the project goals. Alternatively, ranks of locally optimal paths from other initial landscapes will vary considerably (Fig 8B), and in these instances many of the lower-ranked options will clearly have to be excluded from consideration.

### Case study results

**Fig 9:**
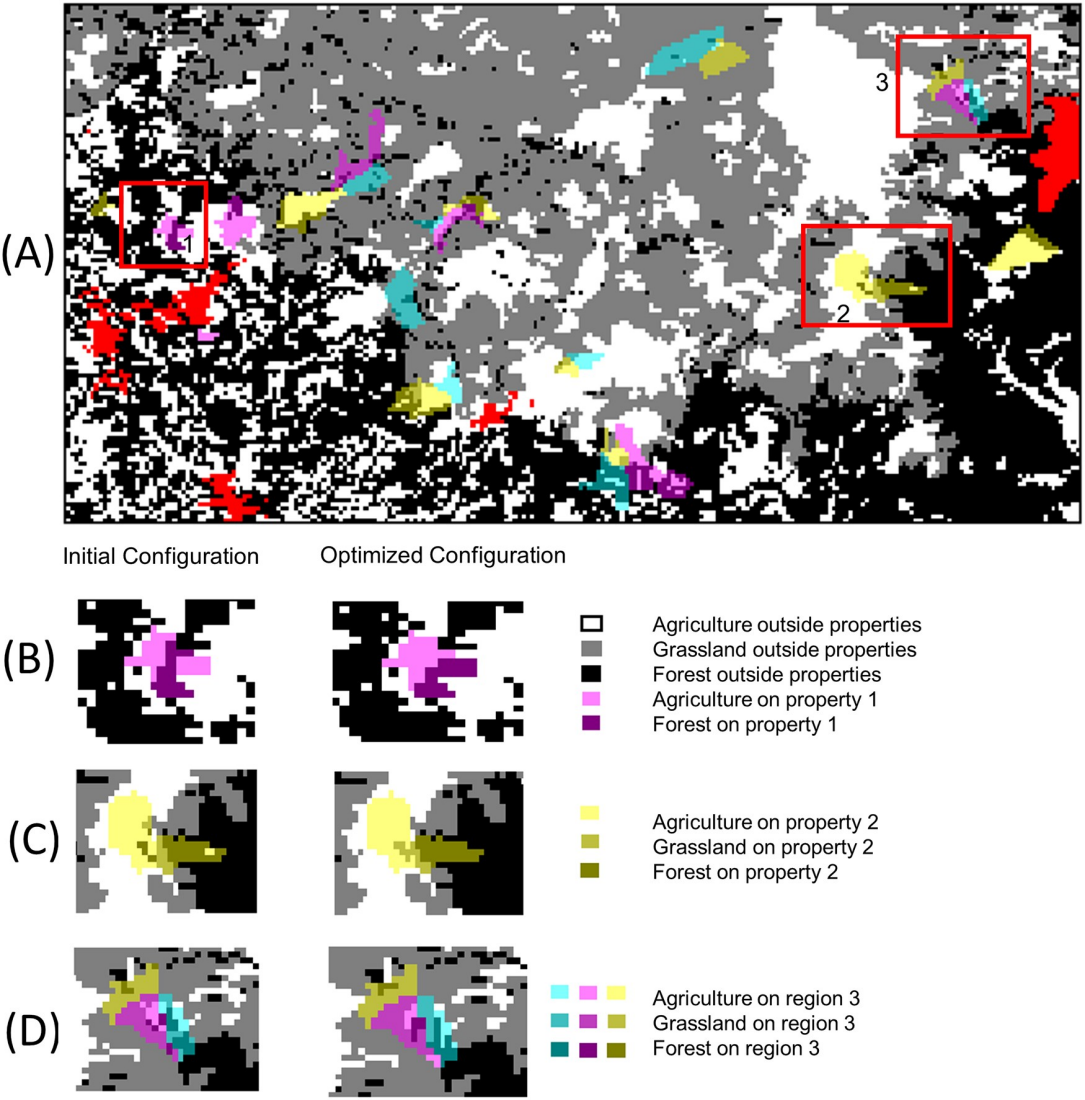
Case study results. (A) Vegetation states of all the parcels on the case study landscape before the algorithm was applied (initial landscape). White parcels are agricultural land, gray parcels are grassland, and the black parcels are forest land. Red parcels are urban and water parcels. The other colors (magenta, cyan, and yellow) overlaid on the map represent the landowner properties (>50 ha in area) that have been registered through INCRA as of November 2015. (B)-(D) Optimal configuration for three highlighted landowner properties on the case study landscape.

The findings from the hypothetical landscape study generally hold for the case study region (Fig 9). That is, on each landowner property the algorithm systematically connects all grassland parcels, then all forest parcels, and finally all agricultural parcels. The parcel exchange maps are not shown for the case study, but the optimal configuration for three landowner properties are shown, and exhibit the single-patch behavior that was also seen in Figs 4 and 5 (Fig 9B–9D).

For the first set of case study simulations (Fig 9) only intra-property parcel exchanges were considered. This assumption resulted in an important observation that was not seen in the simulations on the hypothetical landscapes, and is independent of the choice of the objective function. Specifically, organizing all the parcels into a single patch on a property often breaks up patches in larger regions that encompass the property. This can be observed by noting that the forest patch on the property in Fig 9B was part of a larger forest patch before the optimization (left panel), and then after the optimization the forest patch on the property is now separated from the larger forest patch to the north (right panel). A similar phenomenon can also be observed in Fig 9C and 9D. In Fig 9C, before optimization (left panel), the grassland parcels on the property were part of a larger grassland patch, and after the optimization the grassland patch on the property has been separated from the state-owned grassland to the north. In Fig 9D, note that before the optimization (left panel) the forest land on the center property (magenta) and the east property (cyan) were connected, but when the optimization algorithm was run on the individual property owner’s landscapes, the forested lands on the two properties are now disconnected. Hence, the spatial scale over which optimization must be applied is an important consideration.

**Fig 10:**
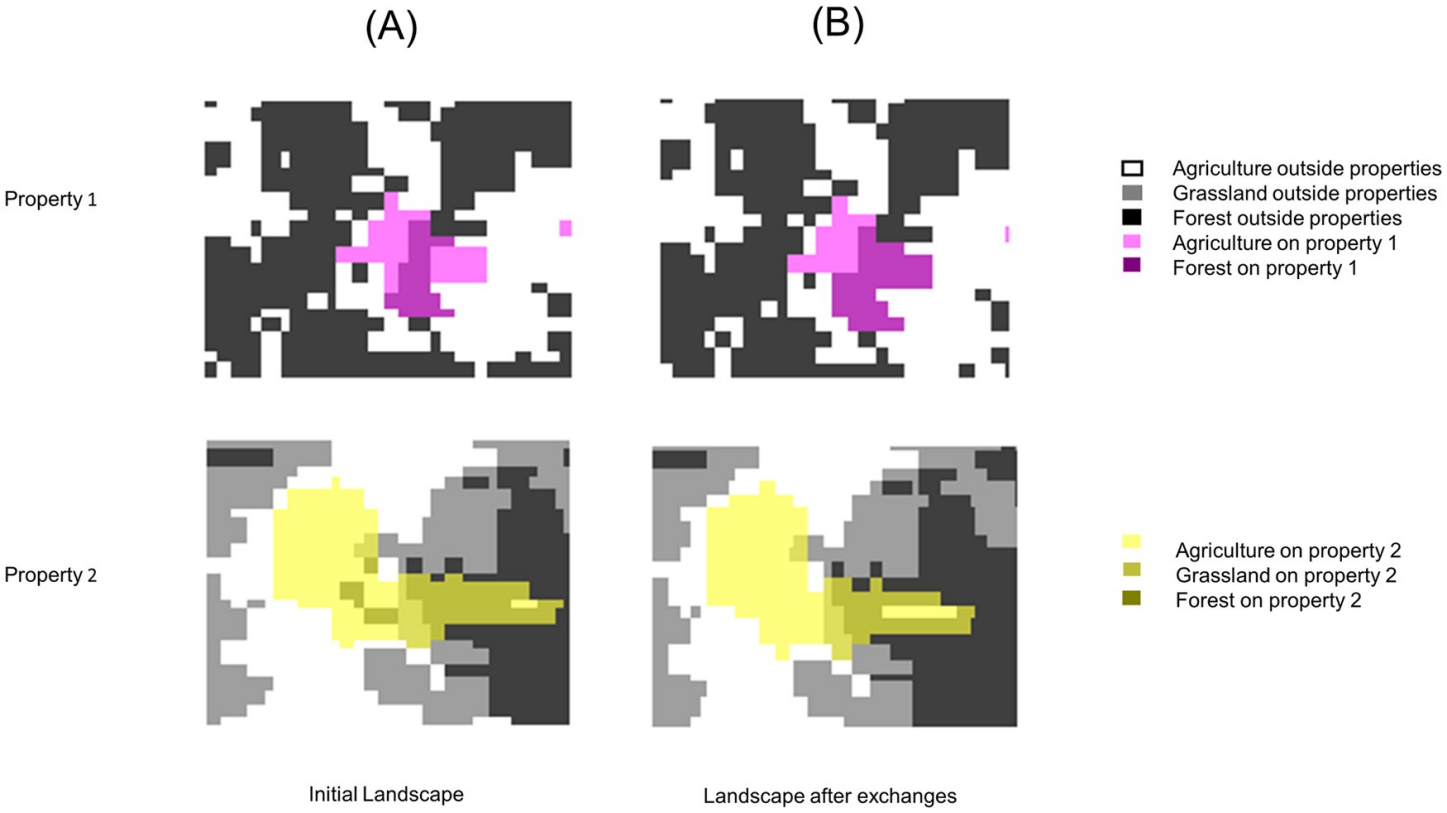
Simulated CRA exchange. A) Initial landscape configurations of property owners 1 (top) and 2 (bottom) (see Fig 9A to see where these properties fit into the landscape). B) Locally optimal landscape configurations after property owner 2 increases their agricultural land by 10 parcels by participating in a CRA-type exchange with property owner 1. In exchange for a negotiated price, property owner 1 agrees to grow 10 parcels of forest on their agricultural land.

In the second set of case study simulations we investigated a CRA-type exchange between two property owners. That is, in this simulation the only allowable parcel exchanges were inter-property exchanges between property owner 1 and property owner 2 (see Fig 9A). In Fig 10 the initial configurations (Fig 10A) and one set of locally optimal configurations (Fig 10B) are shown if, for a negotiated price, property owner 1 decides to exchange an area of agricultural land equivalent to 10 parcels for an area of forested land equivalent to 10 parcels with property owner 2. Note that the algorithm is still making one parcel exchange per iteration, but an automatic stop after 10 iterations is included in the algorithm to account for the agreed upon exchange area. That is, property owner 2 is increasing their agriculture land one parcel at a time and property owner 1 is increasing their forest land one parcel at a time. Note that even though we changed the allowable parcel exchanges to inter-property exchanges in this simulation, our general results are still observed. Most notably, the optimal configuration places the exchanged parcels into the largest existing patch of that type.

## Discussion

We have introduced a landscape optimization framework that provides an additional resource to decision-makers attempting to resolve conservation conflicts through the spatial rearrangement of landscape elements such as what occurs under Brazil’s environmental reserve quota (CRA), and focusing on application to the forest-grassland mosaics in Rio Grande do Sul, Brazil. The framework applies a local optimization approach instead of a global approach, and offers the following advances to the research field. The local search algorithm (i) identifies multiple, viable options of locally optimal landscapes that improve species richness compared to what was sustained on the initial landscape (Figs 4 and 5), (ii) provides a rearrangement schedule to obtain each of the locally optimal landscapes through the aid of step-by-step parcel exchange maps (Figs 6 and 7), and (iii) provides a ranking system to assist the land managers in evaluating the multiple options (Fig 8). Another interesting result is that we have identified instances in which locally optimal landscapes may be preferred to globally optimal landscapes due to the fact they may be easier to implement, more cost-effective, and/or almost as good as a single globally optimal solution (Fig 8).

This work is related to the field of systematic conservation planning, but research in that area is primarily focused on optimal design of nature reserves [14]. Alternatively, here we focused on investigating potential resolutions to conservation conflicts on working lands, i.e., landscapes where conservation mandates must be met in the presence of agricultural lands, such as an individual landowner’s property in Brazil. Hence, conservation planning software such as Marxan, Zonation, and C-plan [15] designed for optimal reserve design are not directly applicable to the problem we have investigated. The methodology we developed also differs from previous studies on working lands [16,17,20] in that local solutions rather than global solutions were sought. One advantage of local optimization approaches is the finding of multiple near-optimal solutions, which can provide a list of options for decision-makers to consider. While some landscape constraints can be mathematically modeled and easily incorporated into the objective function, other constraints will be difficult to quantify, such as those arising from social, socioeconomic, or cultural practices. In these instances, providing a list of options may be helpful. Another advantage of local optimization is its dependence on initial condition data, which can provide a strategy to move forward starting from the current landscape.

The optimization technique employed was a local search algorithm that considered all one-parcel exchanges. Local search algorithms are a subset of local heuristics referred to as standard hill-climbing algorithms [46], which result in local optima and improvements in the objective function at every iteration of the algorithm. The hill-climbing approach enabled step-by-step parcel exchange maps (locally optimal paths) to be produced, which not only ensures that species richness is improved over the course of the rearrangement process, but also provides a strategy to handle budgetary and work force constraints. Optimization algorithms (e.g., Marxan, Marxan with Zones, Zonation, and C-plan [15]) used in similar studies identify a global solution, i.e., an end landscape that satisfies the objective(s) better than any other landscape [16]. The common methods used to obtain globally optimal solutions include exact integer programming methods and/or metaheuristics (e.g., simulated annealing [53] or genetic algorithms [13,17,20]). These algorithms do not require the objective function to improve at each step, and hence do not provide optimal pathways for stepwise re-arrangements to reach a global optimum. In general, local search algorithms do not necessarily reach the global optimum; however, as it was demonstrated in the results shown here, they do have the potential to do so (Figs 4B–4K and 5F–5K).

Another feature of our local optimization algorithm is its flexibility in application. Land state types can easily be added or removed, the type of allowable parcel exchanges can be modified, and parcel size can be changed. Moreover, patchy environments that result from disturbances, such as fire, can easily be handled by supplying the algorithm with a new initial condition (corresponding to the disturbed landscape), and simply proceeding with the optimization from the new initial condition.

Here we focused on optimization when the proportions of agricultural lands, forest, and grassland are conserved during land state changes, such as under the Brazilian CRA. However, this approach could be adapted for other applications. For example, if the project goal was to expand agricultural lands at minimal cost to species richness, or restore native vegetation in a way that maximizes species richness, the methodology here can be adapted to these situations by changing step 2 of Fig 2 and possibly changing the objective function.

Our approach makes several simplifying assumptions, some of which were discussed in Methods. Although the algorithm can determine global optima under some circumstances (Figs 4B–4K and 5F–5K), this is not always the case. Since local search algorithms require an improvement at each iteration, they are more likely to get trapped in local optima (see Fig 5B–5E) than other algorithms [46], and therefore are less likely to reach global optima. Secondly, it was assumed that agricultural plots are subject to SARs (*c*_*Ag*_ = 0.05*c*_*G*_) such that larger agriculture patches will contain more species. However, it is possible smaller, more evenly dispersed agriculture plots over the landscape may be optimal with respect to overall biodiversity [54]. Other improvements could be made to the objective function. For example, differences in reestablishment times and financial costs of land state changes could be included using multi-objective optimization, or by weighting the two disparate outcomes in a single utility function expressing both ecological and economic outcomes. As with any useful planning tool, collaboration with stakeholders and experts in local ecological systems will be required to establish an objective function that ensures the relevant local features are adequately represented.

The first set of simulations in the case study of the *Araucaria*-*Campos* mosaic (Fig 9) suggests that the spatial scale is an important issue in landscape management: what is optimal at the property level is not necessarily optimal at the landscape level. We expect this finding to be robust to changes in the objective function. The results suggest that policies enforced at the property level may not achieve the desired result of improved biodiversity at the landscape level. This interpretation suggests that conservation polices that are solely based on landowners satisfying a given legal reserve percentage on their property should be revised to consider landscape-level connectivity.

The second set of simulations in the case study (Fig 10) were run to further demonstrate the applicability and flexibility of local search algorithms in landscape planning problems. We have mentioned throughout this paper that the Brazilian government requires landowners to satisfy a certain legal reserve (e.g., at least 20% of their land consists of native vegetation). However, the recently introduced CRA in the Brazilian Forest Code allows two property owners to participate in a parcel exchange program that allows one landowner to violate the legal reserve requirement if another landowner (that has a surplus) agrees to conserve an equal amount of land. Fig 10 provides a hypothetical example of two landowners exchanging 10 parcels of agriculture land for 10 parcels of forest land, and provides an idea of how CRA-type exchanges can be modeled, and how the model could be used to assist in the transaction between two willing parties. Of course, an office that connects the willing parties and runs the simulations will need to be established, and this is discussed further in the following paragraph. The primary take-home message from the results associated with Fig 10 is that our algorithm can handle very specific restrictions that a project may require. In this case the restriction placed on the algorithm was to only consider inter-property exchanges between two specified properties; however, due to the logical nature of mathematical programming, restricting and adjusting the local search space to very specific user preferences is relatively straightforward.

With the growing inclusion of land offsetting programs into legislation at all levels of governance, such as the environmental reserve quota (CRA) in the Brazilian Forest Code [34], predicting the biodiversity consequences of changes in vegetative states and trying to guide land use changes to preserve biodiversity is of the utmost importance. Authorities in most countries cannot mandate parcel exchanges that would adhere to the increasing minimum path predicted by ours or any other local search algorithm. However, conceivable policy mechanisms that could nonetheless promote exchanges that move the landscape to the local optimum include tax and subsidy mechanisms. One policy mechanism we propose that could potentially be used to move landowners along an increasing minimum path is to create an office (government, NGO, farmer-led, or other third-party) that is responsible for establishing an appropriate objective function for the region and running the optimization simulations. The office would then share information amongst landowners about feasible land exchanges that increase the mosaic richness index, and offer incentives to landowners who participate in the project. Alternatively, taxes and subsidies could be used to incentivize or disincentivize certain exchanges. For instance, a pair of landowners who wish to conduct an exchange that reduces connectivity could pay a tax to conduct that exchange, while exchanges that promote connectivity could be subsidized. Further research could study pathways under this approach and compare them to the paths predicted by our local search algorithm.

Planning and management strategies that apply to the special characteristics of regional landscapes are also needed [2,55,56]. In particular, a high priority should be placed on the establishment of local land-use management policies in lower-income countries, where a vast majority of the world’s biodiversity hotspots can be found [22,57]. Our analysis shows that local optimization approaches can provide multiple possible solutions that are almost as good as the single globally optimal solution in terms of satisfying the objective function. However, these multiple solutions may differ from one another in other ways, and these differences may matter to stakeholders in ways that may be difficult to quantify with an objective function, such as with respect to equity or cultural norms. Local optimization also provides a pathway to move from the current land state to alternative land states with higher species richness. We conclude that using local optimization in tandem with global optimization approaches shows potential as an effective landscape management tool to protect terrestrial biodiversity.

## Supporting information

S1 Appendix Attached separately. (PDF)

S2 Appendix Attached separately. (PDF)

S1 File Grassland data. Attached Separately.
(XLSX)

S2 File Forest data. Attached Separately.
(XLSX)
