# Supplementary material for: A local optimization framework for addressing conservation conflicts in mosaic ecosystems
Source: PLoS One. 2019 May 31;14(5):e0217812. doi: 10.1371/journal.pone.0217812 (PMC6544304; doi:10.1371/journal.pone.0217812)
Supplement: S1 Appendix — (PDF) [file pone.0217812.s001.pdf]

Optimizing Solutions to Conservation Conflicts in Mosaic Ecosystems – S1 Appendix

Shane Nowack, Chris T. Bauch, and Madhur Anand

Journal - PLoS One
Corresponding author - Shane Nowack. University of Guelph, School of Environmental Sciences, Guelph, ON, N1G 2W1, Canada. spnowack@gmail.com

The purpose of this supplementary material is to mathematically show that the objective function stated in the methods section, subject to the imposed assumptions, takes on its absolute maximum value (global optimum) whenever the parcels of each vegetative state (Ag, F, and G) are collected into a single patch. Recall that the objective function was a measure of species biodiversity and defined as

$$B = \sum_{L=1}^{Q} B_L, \tag{1}$$

where

$$\begin{aligned} B_L &= \sum_{k|P_k \epsilon D_L} c_j \left(\sum_{k|P_k \epsilon D_L} A x_{k,j} \right)^{z_j} \\ &= \sum_{k|P_k \in D_L} c_j A_L^{z_j} \end{aligned}$$

was the species biodiversity that each patch is modeled to sustain. An important observation is that, with this formulation, the biodiversity score is strictly determined by the number of parcels per patch. This can be seen by rewriting B_L in a different form. Let

a_L represent the number of parcels in agriculture patch L, f_L represent the number of parcels in forest patch L, and g_L represent the number of parcels in grassland patch L. Regardless of the spatial arrangement of a patch consisting of j_L parcels, where $j \in \{a, f, g\}$ the biodiversity score of that patch will be

$$\begin{aligned} B_L =& j_L c_j (A * j_L)^{z_j} \\ =& A^{z_j} c_j j_L^{z_j+1}. \end{aligned}$$

Recall that $A_L = A * j_L$, A is the area of each uniform square parcel on the landscape, and c_j and z_j are the parameters defining the species-area relationships for a patch in vegetative state j, and were determined from the empirical data. Next, the terms of B are rearranged so that the biodiversity scores of the patches of each vegetative type are grouped together. That is

$$B = c_{Ag} A^{z_{Ag}} \left(\sum_{L=1}^{Q_a} (a_L)^{z_{Ag}+1} \right) + c_F A^{z_F} \left(\sum_{L=1}^{Q_f} (f_L)^{z_F+1} \right) + c_G A^{z_G} \left(\sum_{L=1}^{Q_g} (g_L)^{z_G+1} \right), \tag{2}$$

where Q_a, Q_f, and Q_g represent the total number of agriculture, forest, and grassland patches on the landscape, respectively. The model constraint that the number of parcels in each vegetative state are assumed to be equal can also be rewritten:

$$\begin{aligned} \sum_{L=1}^{Q_a} a_L =& M/3 \\ \sum_{L=1}^{Q_f} f_L =& M/3 \\ \sum_{L=1}^{Q_g} g_L =& M/3, \end{aligned} \tag{3}$$

where M is the total number of parcels on the landscape. Since the total biodiversity score and the constraints can both be decoupled into their individual vegetation types, the maximum of (1) can be determined by maximizing each of the terms in (2) subject to their corresponding constraint in (3). Thus, first, we seek to identify the number of patches, Q_a, that maximize

$$c_{Ag} A^{z_{Ag}} \sum_{L=1}^{Q_a} (a_L)^{z_{Ag}+1} \tag{4}$$

subject to

$$\sum_{L=1}^{Q_a} a_L = M/3, \tag{5}$$

where $0 \leq a_L \leq M/3 \; \forall$ L.

Claim. (4), when constrained by (5), attains its maximum when $Q_a = 1$.

Proof of claim. (By induction).

Base case. We show that (4) is larger when $Q_a = 1$, and hence all parcels are in a single patch, than when $Q_a = 2$ and the parcels are divided up into two patches, regardless of how many parcels per patch.

Proof of base case. Since c_{Ag} and $A^{z_{Ag}}$ are the same for each Q_a, showing

$$(a_1 + a_2)^{z_{Ag}+1} \geq a_1^{z_{Ag}+1} + a_2^{z_{Ag}+1} \tag{6}$$

will prove the base case. The veracity of the inequality in (6) will depend on the value of z_{Ag}. In the application discussed in the main text, $z_j = 0.51$ was used for $j \in \{Ag, F, G\}$, and thus, $z_j + 1 > 1 \; \forall \; j$. Therefore, Bernoulli's inequality (Carothers, 2000) can be applied

to the left-hand side of (6), yielding

$$(a_1 + a_2)^{z_{Ag}+1} \geq a_1^{z_{Ag}+1} + a_2 a_1^{z_{Ag}}, \tag{7}$$

and by the commutativity of addition, Bernoulli's inequality also implies

$$(a_1 + a_2)^{z_{Ag}+1} \geq a_2^{z_{Ag}+1} + a_1 a_2^{z_{Ag}}. \tag{8}$$

Note that when $a_1 \geq a_2$

$$\begin{aligned} a_1 &\geq a_2 \Rightarrow \\ a_1^{z_{Ag}} &\geq a_2^{z_{Ag}} \Rightarrow \\ a_2 a_1^{z_{Ag}} &\geq a_2^{z_{Ag}+1}, \end{aligned} \tag{9}$$

and that when $a_2 \geq a_1$

$$\begin{aligned} a_2 &\geq a_1 \Rightarrow \\ a_2^{z_{Ag}} &\geq a_1^{z_{Ag}} \Rightarrow \\ a_1 a_2^{z_{Ag}} &\geq a_1^{z_{Ag}+1}. \end{aligned} \tag{10}$$

Applying (9) to the right-hand side of (7) and (10) to the right-hand side of (8) shows that, $\forall\ a_1, a_2 \geq 1$ and $z_{Ag} \geq 0$,

$$(a_1 + a_2)^{z_{Ag}+1} \geq a_1^{z_{Ag}+1} + a_2^{z_{Ag}+1}. \tag{11}$$

This completes the proof of the base case.

Inductive step. Given that

$$\left(\sum_{L=1}^{N} a_L\right)^{z_{Ag}+1} \geq \sum_{L=1}^{N} a_L^{z_{Ag}+1}, \tag{12}$$

$\forall\ a_L$, show that

$$\left(\sum_{L=1}^{N+1} a_L\right)^{z_{Ag}+1} \geq \sum_{L=1}^{N+1} a_L^{z_{Ag}+1}. \tag{13}$$

Proof of inductive step. Starting from the left-hand side of (13),

$$\begin{aligned}
\left(\sum_{L=1}^{N+1} a_L\right)^{z_{Ag}+1} &= \left(\sum_{L=1}^{N} a_L + a_{N+1}\right)^{z_{Ag}+1} \\
&\geq \left(\sum_{L=1}^{N} a_L\right)^{z_{Ag}+1} + (a_{N+1})^{z_{Ag}+1} \qquad \text{(base case)} \\
&\geq \sum_{L=1}^{N+1} a_L^{z_{Ag}+1} \qquad \text{(inductive assumption)},
\end{aligned}$$

completing the proof of the inductive step, and the claim that $Q_a = 1$ is optimal under the given assumptions. q.e.d. The proofs for the forest patches and grassland patches follow identically. We conclude that any landscape that has the parcels of each vegetative patch arranged into a single, connected patch is a globally optimal arrangement. It is important to reiterate that this result is dependent on the assumptions of model, namely the form of the objective function, and the values of the species-area parameters. Specifically, if all assumptions were the same except $z_j + 1 < 1$, Bernoulli's inequality would be reversed, and organizing all the parcels into one-parcel patches would be optimal.

S1 Appendix References

Carothers NL (2000) Real analysis. Cambridge University Press, Cambridge
