# Supplementary material for: A local optimization framework for addressing conservation conflicts in mosaic ecosystems
Source: PLoS One. 2019 May 31;14(5):e0217812. doi: 10.1371/journal.pone.0217812 (PMC6544304; doi:10.1371/journal.pone.0217812)
Supplement: S2 Appendix — (PDF) [file pone.0217812.s002.pdf]

A local optimization framework for addressing conservation conflicts in mosaic ecosystems – S2 Appendix- MATLAB CODE

Shane Nowack, Chris T. Bauch, and Madhur Anand

Journal – PLoS One

Corresponding author – Shane Nowack. University of Guelph, School of Environmental Sciences, Guelph, ON, N1G 2W1, Canada. spnowack@gmail.com

1) **Main Algorithm (local search algorithm). This algorithm call the objective function which is given in 2) below.**

```
%Local search algortithm for determining local optima, starting from a
%given initial condition
% Written by Shane Nowack
clear
M=36; % number of parcels
N=3; % number of vegetative states
A_L=1000000; %Area of landscape
A_p=A_L/M; %area of each parcel
load big_dc_6 %this is matrix consisting of distance each parcel is from every other
%Need to run Parcel_distance.m first.  D_c is the name of the matrix.
load initial_condition_fig_2B%intial condition for Figure 2.  x is the name of IC.
% load paper_fig3 %initial condition for Figure 3
%load initial_condition_fig_3B
f_2=x;
obj=zeros((1/3)*M^2,1);
count_it=1;
old_f2=f_2;
%hj=size(d)
curr_x=objective_function(f_2,D_c);  %call to obejcetive function that models biodiversity
%new_obj=-inf;
new_obj=curr_x;
max_itqr=100;
v=1;
w=2;
path_it=1;
old_path_it=1;
%Loop that finds the optimal one parcel exchange, makes the change, and
%ensures the biodiversity is improved on the new landscape.
```

```
while(new_obj<curr_x || w>1)
x_new=f_2;
x_A=find(x_new(1:M)==1);%current parcels in Ag state
x_B=M+find(x_new(M+1:2*M)==1); %current parcels in F state
x_C=2*M+find(x_new(2*M+1:3*M)==1); %current parcels in G state
counter=1;
decoder=zeros((1/3)*M^2,2);
i=1;
%%%%%%%%%%%%%%%%%%%%%%%%%%%%%%%%%%%%%%%%%%%%%%%%%%%
%Loops that test all one-parcel exchanges%
%%%%%%%%%%%%%%%%%%%%%%%%%%%%%%%%%%%%%%%%%%%%%%%%%%%
        for i=1:length(x_A)
        x_new=f_2;
        x_new(x_A(i))=f_2(x_A(i)+M); %makes first white square black
        x_new(x_A(i)+M)=f_2(x_A(i));
        for j=1:length(x_B)
        x_new(x_B(j)-M)=f_2(x_B(j));
        x_new(x_B(j))=f_2(x_B(j)-M);
        obj(counter)=objective_function(x_new,D_c);
        decoder(counter,:)=[x_A(i) x_B(j)];
        x_new(x_B(j)-M)=f_2(x_B(j)-M);
        x_new(x_B(j))=f_2(x_B(j));
        counter=counter+1;
        end
    end
 for i=1:length(x_A)
       x_new=f_2;
       x_new(x_A(i))=f_2(x_A(i)+2*M);
       x_new(x_A(i)+2*M)=f_2(x_A(i));
        for j=1:length(x_C)
        x_new(x_C(j)-2*M)=f_2(x_C(j));
        x_new(x_C(j))=f_2(x_C(j)-2*M);
        obj(counter)=objective_function(x_new,D_c);
        decoder(counter,:)=[x_A(i) x_C(j)];
        x_new(x_C(j)-2*M)=f_2(x_C(j)-2*M);
        x_new(x_C(j))=f_2(x_C(j));
        counter=counter+1;
        end
 end
   for i=1:length(x_B)
       x_new=f_2;
       x_new(x_B(i))=f_2(x_B(i)+M);
        x_new(x_B(i)+M)=f_2(x_B(i));
        for j=1:length(x_C)
        x_new(x_C(j)-M)=f_2(x_C(j));
        x_new(x_C(j))=f_2(x_C(j)-M);
```

```
        obj(counter)=objective_function(x_new,D_c);
        decoder(counter,:)=[x_B(i) x_C(j)];
        x_new(x_C(j)-M)=f_2(x_C(j)-M);
        x_new(x_C(j))=f_2(x_C(j));
        counter=counter+1;
        end
    end
%%%%%%%%%%%%%%%%%%%%%%%%%%%%%%%%%%%%%%%%%%%%%%
%%%%%%%%%%
min_obj=min(obj); %min of objective after all one-parcel exchanges
%have been considered
%%%%%%%%%%%%%%%%%%%%%%%%%%%%%%%%%%%%%%%%%%%%%%
%%%%%%%%%%
% Identify minimizers and makes the appropriate exchange
%%%%%%%%%%%%%%%%%%%%%%%%%%%%%%%%%%%%%%%%%%%%%%
%%%%%%%%%%
if (min_obj<new_obj)
    find_1=find(obj==min_obj) %finds which parcel exchanges result in optimal biodiversity
    L_1=length(find_1)
    next_up=zeros(3*M,L_1); %preallocating matrix to store all minimizers
    for i=1:L_1
    find_x=find_1(i)
    f_2=old_f2;
    swap_coord=mod(decoder(find_x,:),M) %coordinates needed to be swapped
        if (swap_coord(1)==0)
        swap_coord(1)=M;
        elseif (swap_coord(2)==0)
        swap_coord(2)=M;
        end
        g=f_2;
    if (find_x<=(M^2)/9)
    f_2(swap_coord(1))=g(swap_coord(1)+M);
    f_2(swap_coord(1)+M)=g(swap_coord(1));
    f_2(swap_coord(2))=g(swap_coord(2)+M);
    f_2(swap_coord(2)+M)=g(swap_coord(2));
    elseif (find_x<=(2*(M^2))/9 && find_x>(M^2)/9)
    f_2(swap_coord(1))=g(swap_coord(1)+2*M);
    f_2(swap_coord(1)+2*M)=g(swap_coord(1));
    f_2(swap_coord(2))=g(swap_coord(2)+2*M);
    f_2(swap_coord(2)+2*M)=g(swap_coord(2));
    else
    f_2(swap_coord(1)+M)=g(swap_coord(1)+2*M);
    f_2(swap_coord(1)+2*M)=g(swap_coord(1)+M);
    f_2(swap_coord(2)+M)=g(swap_coord(2)+2*M);
    f_2(swap_coord(2)+2*M)=g(swap_coord(2)+M);
    end
```

```
    next_up(1:3*M,i)=f_2;   %new landscapes
    if (count_it==1 && i==1)
    switch_hist=swap_coord;
    end
    next_up(3*M+1,i)=swap_coord(1);%ghju=size(next_up)
    next_up(3*M+2,i)=swap_coord(2);
    end
    curr_x=objective_function(old_f2,D_c);  %compare objective function value
    new_obj=objective_function(next_up(1:3*M,1),D_c);  %before and  after the exchange
else
    curr_x=objective_function(old_f2,D_c);
    new_obj=objective_function(f_2,D_c);
end
%%%%%%%%%%%%%%%%%%%%%%%%%%%%%%%%%%%%%%%%%%%%%%%%%%%%%
%%%%%%%%%%%%%%%%%%%%%%%%%%%%%%%%%%%%%
% The remaining code stores the information about what parcels were exchanged, and
% manages the ties to ensure all paths from each minimizer are followed.
%%%%%%%%%%%%%%%%%%%%%%%%%%%%%%%%%%%%%%%%%%%%%%%%%%%%%
%%%%%%%%%%%%%%%%%%%%%%%%%%%%%%%%%%%%%%%%%%
    if (count_it==1)
        w=2;
        the_main=next_up;
        mm=1
    else
        [v,w]=size(the_main); %candidates left in queue
    end
     if (w==1 && curr_x>new_obj) %if no tie on first it for example
        the_main=next_up;
        mm=2
     end

     if (w>1 && curr_x>new_obj && count_it>1)
            the_main=[next_up the_main(:,2:end)];
            mm=3
     end
        if(count_it==1)
        switch_list=[switch_hist L_1]
        elseif (count_it>1 && curr_x>new_obj)
        switch_list=[switch_list; the_main(3*M+1,1) the_main(3*M+2,1) L_1]
        end
        [beta_row,beta_col]=size(switch_list)
        thresh_h=11; %minimal number of exchanges required to reach a local optima plus 1
        if (beta_row==thresh_h && curr_x>new_obj) %if thresh_h parcel exchanges have
happened and still improving quit.  Go back to better options
            the_main=the_main(:,L_1+1:end); %if an error is reached here this means the local opt
can't be achieved with threshold conditions
```

```
            curr_x=objective_function(the_main(1:3*M,1),D_c);
            new_obj=curr_x-1;
            side_no=find(switch_list(1:end-1,3)>1); %same as above
            side_index=max(side_no);
            new_side_index= switch_list(side_index,3)-1;
            switch_list=[switch_list(1:side_index-1,:); the_main(3*M+1,1) the_main(3*M+2,1)
new_side_index]
        end
        if (w>1 && curr_x<=new_obj && count_it>1)
            side_no=find(switch_list(:,3)>1);
            side_index=max(side_no);
            new_side_index= switch_list(side_index,3)-1;
            beta{path_it}=switch_list(:,1:2); %stores all parcel exchanges for each minimal path.
This
            %output is what was used to construct figures.
            the_main=the_main(:,2:end);
            switch_list=[switch_list(1:side_index-1,:); the_main(3*M+1,1) the_main(3*M+2,1)
new_side_index]
            path_it=path_it+1;
            curr_x=land_use_objective_bauch_6_fast(the_main(1:3*M,1),D_c);
            new_obj=curr_x-1;
            mm=4
        end
        if (count_it==1 && L_1==1)
            w=1;
            mm=5
        end
        if (w==1 && curr_x<=new_obj)
            beta{path_it}=switch_list(:,1:2);
            path_it=path_it+1;
            mm=6
        end
        [beta_row,beta_col]=size(switch_list)
        old_f2=the_main(1:3*M,1); %the current landscape
        f_2=the_main(1:3*M,1);
        size_check=size(the_main)
        count_it=count_it+1
end
```

2) Objective function algorithm.

```
    %Objective function that model biodiversity.  Called by local_search.m.

    %The local search algorithm is written as a minimization problem so the

    %objective function is the negative of biodiversity
```

```
function F = objective_function(x,D_c)


A_L=1000000; %area of landscape (100 Ha)

M=36; % number of parcels

A_p=A_L/M; % area of each parcel


%%%%%%%%%%%%%%%%%%%%%%%%%%%%%%%%%%%%%%%%%%%%%
%%%%%%%%%%%

% Parameters of objective function determined from SAR %

z_G=.51;

c_G=19.95;

z_F=z_G;

c_F=.5*c_G;

z_A=z_G;

c_A=.05*c_F;

%%%%%%%%%%%%%%%%%%%%%%%%%%%%%%%%%%%%%%%%%%%%%
%%%%%%%%%%%%%%%

% Code that determines how many ag parcels are in each patch

%%%%%%%%%%%%%%%%%%%%%%%%%%%%%%%%%%%%%%%%%%%%%
%%%%%%%%%%%%%%%%

M_count=1:1:M;

list_ag=find(x(1:M)==1);

ag_mat_1=bsxfun(@times,x(1:M)',D_c);

next_ag=list_ag(1);

new_list_ag=list_ag';

patch_ag=zeros(M/3,1);

counter=0;

wait_list=[];
```

```
pat_counter=1;

while isempty(new_list_ag)==0

    %ag_mat_1(:,queue)=0;

    counter=counter+1; %counts parcels in patch

    new_list_ag(new_list_ag==next_ag)=[]; %removes next_ag parcel from ag parcel list

    %queue=find(ag_mat_1(next_ag,:)==1); %finds parcels that are neighbors of next_ag

    queue_2=ag_mat_1(next_ag,:)==1;

    queue=M_count(queue_2);

    %if isempty(queue)==0

    ag_mat_1(:,next_ag)=0; %sets next_ag column to 0 for all parcels in queue (to ensure not
to count parcel twice)

    ag_mat_1(:,queue)=0;

    %end

    W=length(queue);

    if W>1

    wait_list=[queue(2:end) wait_list];

    end

    if isempty(queue)==1 && isempty(wait_list)==1

        patch_ag(pat_counter)=counter;

        counter=0;

        pat_counter=pat_counter+1;

        if isempty(new_list_ag)==0

        next_ag=new_list_ag(1);

        end

    elseif isempty(queue)==1 && isempty(wait_list)==0

        next_ag=wait_list(1);

        wait_list(1)=[];

    elseif isempty(queue)==0
```

```
        next_ag=queue(1);

    end

end

%%%%%%%%%%%%%%%%%%%%%%%%%%%%%%%%%%%%%%%%%%%%%
%%%%%%%%%%%%%%%%%%%

% Code that determines how many forest parcels are in each patch

%%%%%%%%%%%%%%%%%%%%%%%%%%%%%%%%%%%%%%%%%%%%%
%%%%%%%%%%%%%%%%%%%%

list_fo=find(x(M+1:2*M)==1);

fo_mat_1=bsxfun(@times,x(M+1:2*M)',D_c);

next_fo=list_fo(1);

new_list_fo=list_fo;

patch_fo=zeros(M/3,1);

counter=0;

wait_list=[];

pat_counter_2=1;

while isempty(new_list_fo)==0

    counter=counter+1; %counts parcels in patch

    new_list_fo(new_list_fo==next_fo)=[]; %removes next_ag parcel from ag parcel list

    queue_2=fo_mat_1(next_fo,:)==1;

    queue=M_count(queue_2);

    fo_mat_1(:,next_fo)=0; %sets next_ag column to 0 for all parcels in queue (to ensure not
to count parcel twice)

    fo_mat_1(:,queue)=0;

    %end

    W=length(queue);

    if W>1

    wait_list=[queue(2:end) wait_list];
```

```
    end
    if isempty(queue)==1 && isempty(wait_list)==1
        patch_fo(pat_counter_2)=counter;
        counter=0;
        pat_counter_2=pat_counter_2+1;
        if isempty(new_list_fo)==0
        next_fo=new_list_fo(1);
        end
    elseif isempty(queue)==1 && isempty(wait_list)==0
        next_fo=wait_list(1);
        wait_list(1)=[];
    else
        next_fo=queue(1);
    end
end
%%%%%%%%%%%%%%%%%%%%%%%%%%%%%%%%%%%%%%%%%%%%%%%%%%
%%%%%%%%%%%%%%%%%%%%%%%%%%%
% Code that determines how many grassland parcels are in each patch.
%%%%%%%%%%%%%%%%%%%%%%%%%%%%%%%%%%%%%%%%%%%%%%%%%%
%%%%%%%%%%%%%%%%%%%%%%%%%%%
list_gr=find(x(2*M+1:3*M)==1);
gr_mat_1=bsxfun(@times,x(2*M+1:3*M)',D_c);
next_gr=list_gr(1);
new_list_gr=list_gr;
patch_gr=zeros(M/3,1);
counter=0;
wait_list=[];
pat_counter_3=1;
```

```
while isempty(new_list_gr)==0
    counter=counter+1; %counts parcels in patch
    new_list_gr(new_list_gr==next_gr)=[]; %removes next_ag parcel from ag parcel list
    %queue=find(gr_mat_1(next_gr,:)==1); %finds parcels that are neighbors of next_ag
    queue_2=gr_mat_1(next_gr,:)==1;
    queue=M_count(queue_2);
    %if isempty(queue)==0
    gr_mat_1(:,next_gr)=0; %sets next_ag column to 0 for all parcels in queue (to ensure not to count parcel twice)
    gr_mat_1(:,queue)=0;
    %end
    W=length(queue);
    if W>1
    wait_list=[queue(2:end) wait_list];
    end
    if isempty(queue)==1 && isempty(wait_list)==1
        patch_gr(pat_counter_3)=counter;
        counter=0;
        pat_counter_3=pat_counter_3+1;
        if isempty(new_list_gr)==0
        next_gr=new_list_gr(1);
        end
    elseif isempty(queue)==1 && isempty(wait_list)==0
        next_gr=wait_list(1);
        wait_list(1)=[];
    else
        next_gr=queue(1);
    end
```

```
end

%%%%%%%%%%%%%%%%%%%%%%%%%%%%%%%%%%%%%%%%%%%%%
%%%%%%%%%%%

% Code that combines the number of parcels in each patch

% and SARs to define objective function in manuscript.

%%%%%%%%%%%%%%%%%%%%%%%%%%%%%%%%%%%%%%%%%%%%%
%%%%%%%%%%%

B_ag=zeros(pat_counter-1,1);

B_fo=zeros(pat_counter_2-1,1);

B_gr=zeros(pat_counter_3-1,1);

for i=1:pat_counter-1

B_ag(i)=patch_ag(i)*c_A*(patch_ag(i)*A_p)^z_A;

end

for i=1:pat_counter_2-1

B_fo(i)=patch_fo(i)*c_F*(patch_fo(i)*A_p)^z_F;

end

for i=1:pat_counter_3-1

B_gr(i)=patch_gr(i)*c_G*(patch_gr(i)*A_p)^z_G;

end

B_ag_tot=sum(B_ag);

B_fo_tot=sum(B_fo);

B_gr_tot=sum(B_gr);

B=-(B_ag_tot+B_fo_tot+B_gr_tot);

F=B;
```
